# Pinocembrin Protects Against Dextran Sulfate Sodium-Induced Rats Colitis by Ameliorating Inflammation, Improving Barrier Function and Modulating Gut Microbiota

**DOI:** 10.3389/fphys.2019.00908

**Published:** 2019-07-19

**Authors:** Lin Hu, Chao Wu, Zijian Zhang, Mingchang Liu, E. Maruthi Prasad, Yu Chen, Kai Wang

**Affiliations:** ^1^Jiangsu Key Laboratory of Infection and Immunity, Institutes of Biology and Medical Sciences, Soochow University, Suzhou, China; ^2^School of Biology and Basic Medical Sciences, Soochow University, Suzhou, China; ^3^Chinese Academy of Inspection and Quarantine, Beijing, China; ^4^Shenzhen Key Laboratory of Translational Medicine of Tumor, Department of Cell Biology and Genetics, Shenzhen University Health Sciences Center, Shenzhen, China; ^5^Department of Experimental Animals, Zhejiang Academy of Traditional Chinese Medicine, Hangzhou, China; ^6^Institute of Apicultural Research, Chinese Academy of Agricultural Sciences, Beijing, China

**Keywords:** pinocembrin, colitis, tight junction protein, gut microbiota, IBDs

## Abstract

Pinocembrin (PIN) is a natural flavonoid widely found in bee propolis with potent gastrointestinal protective effects. In consequence, PIN has great potential in preventing inflammatory bowel diseases (IBDs) while scant information is available. In this study, a dextran sulfate sodium (DSS)-induced rats ulcerative colitis model (3.5% DSS in drinking water for 7 days) was applied to explore the protective effects of PIN on macroscopic colitis symptoms, inflammation, intestinal epithelial barrier function, and gut microbiota homeostasis. While DSS-treated rats showed severe colitis clinical symptoms and histological changes (colonic pathological damages and intestinal goblet cells loss), pre-administration of PIN (5 and 10 mg/kg, *p.o.*) for a week alleviated these symptoms. Pre-administration of PIN also suppressed the pro-inflammatory gene expressions and improved tight junction functions of colonic epithelial cells. Additionally, PIN administration reversed DSS-induced short chain fatty acid loss, and improved the gut microbial diversity assessed by 16S rRNA phylogenetic sequencing. Overall, our results suggest a wide spectrum of protective effects of PIN in preventing IBDs.

## Introduction

Ulcerative colitis (UC) and Crohn disease (CD) are major forms of the non-specific chronic inflammatory bowel diseases (IBD) with unknown etiology. Studies suggest that main disease segments of UC are the colon/rectum while those of CD are terminal ileum or colon (Neurath, 2017). The consensuses of IBD are several factors, including genetic predispositions, dysfunctional immunity, intestinal barrier dysfunction, and environmental risk factors. In addition, it has been reported widely that the intestinal microbiota plays a vital role in mediating the detrimental factors associated with IBD at different stages (Azad et al., 2018). The intestinal microbiome plays an important role during IBD, and the literature showed characteristic dysbiosis in patients suffered from UC, CD, and pouchitis (Lane et al., 2017). Several therapies have been developed for the management of IBD, for instance using anti-inflammatory (such as sulfasalazine or corticosteroids) and immunosuppressive agents (azathioprine) showed novel biological benefits. However, the applications of these drugs have adverse effects during a long period treatment accompany with high relapse rates (Moura et al., 2015).

**FIGURE 1:**
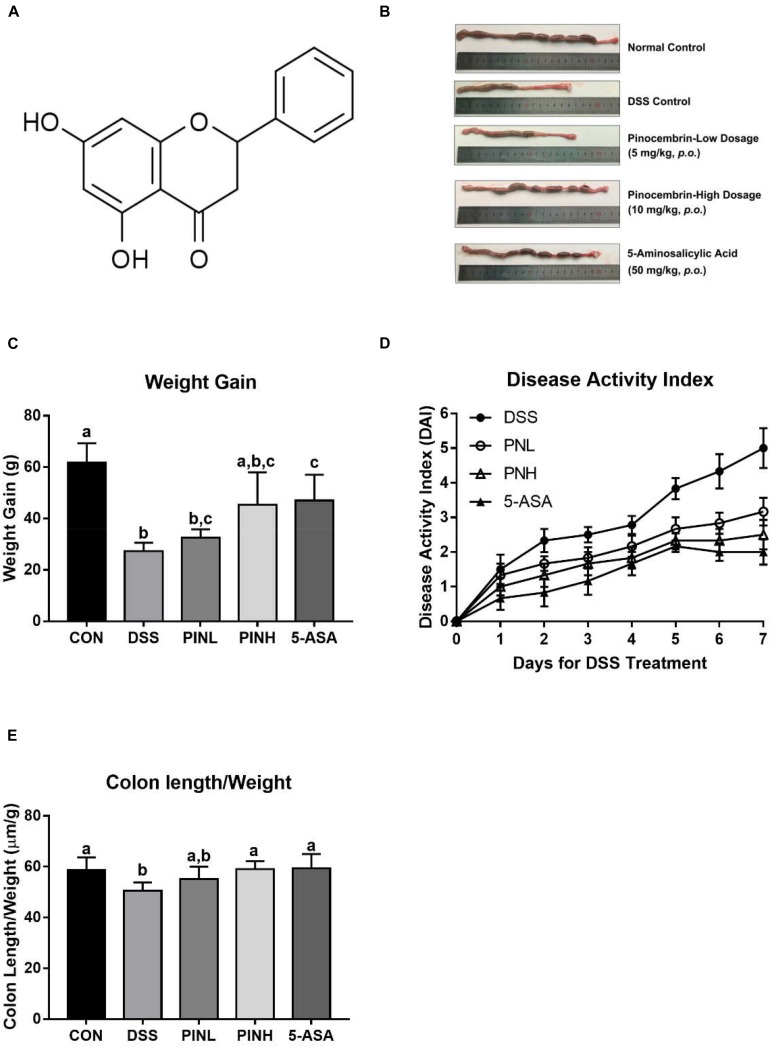
Pinocembrin alleviated colitis symptoms induced by DSS in rats. Pinocembrin (PIN) low dose (PINL 5 mg/kg b.w.), PIN high dose (PINH 10 mg/kg b.w.) **(A)** Chemical structure of Pinocembrin (5,7-dihydroxy flavanone). **(B)** Representative photograph showed macroscopic colon damage from each group. **(C)** Body-weight gain among groups after DSS administration. **(D)** Disease activity index (DAI) values. **(E)** Colon length/body weight. The data represent the mean ± SD of six rats in each group. Means with different letters are significantly different (*p* < 0.05).

Recent days UC and CD have become a heavy burden for public health so there novel and alternative approaches are warranted for IBD patients (Cao et al., 2018). The epidemiological evidence suggests that increasing the diet intake of flavonoids rich fruits and vegetables is associated with low risk of IBD (Ananthakrishnan, 2015; Martin and Bolling, 2015). Pinocembrin (5,7-dihydroxy flavanone, PIN, Figure 1A) is one of a well-studied flavonoid widely found in many natural products, like *Piperaceae* family plants (more than 1950 species plants) (Rasul et al., 2013). PIN was reported to possess various pharmacological properties, including anti-oxidative, anti-inflammatory, neuro-protective and anti-carcinogenic activities (Lan et al., 2016). It is also widely recognized as a key bioactive constitute of bee propolis (Peng et al., 2012). Our research group showed recently that propolis a potent gastrointestinal protector (Wang et al., 2017, 2018). Nevertheless, the effects of PIN against IBD has not been investigated and the mechanisms underlying the activity of PIN remain unknown.

The aim of the present study was to investigate protective effect of pinocembrin (PIN) on colitis severity symptoms, anti-inflammatory activities, restoring on intestinal barrier function and modulating gut microbial populations using a DSS-induced rats colitis model. Our working hypothesis is that PIN alleviated DSS-induced experimental colitis severity symptoms by its anti-inflammatory activities, restoring on intestinal barrier function as well as modulating gut microbial populations.

## Materials and Methods

### Chemical and Regents

PIN (purity >99%) was purchased from Biopurify Phytochemicals Ltd. (Chengdu, China); and DSS (M.W. 36–50 kDa) was purchased from MP Biomedicals (Irvine, CA, United States). All other reagents were obtained from Sangon Biotechnology (Shanghai, China) or as indicated in specified methods.

### Animals and Acute Colitis Induction

Male Sprague Dawley rats (30 rats, 6 weeks old, 190–220 g) were housed in the Animal Experimental Center of the Zhejiang Institute of Traditional Chinese Medicine, in the SPF environment which following standard experimental protocols and approved from Animal Ethics Committee of Institute of Apicultural Research, Chinese Academy of Agricultural Sciences. All rats are fed by strand lab chow (Xietong Biotechnology, Nanjing, China) and water *ad libitum* and were maintained on a 12 h light/dark cycle (21 ± 2°C with a relative humidity of 45 ± 10%). After 3 days acclimatization, experiment was started (7 days of drug pretreatment following by 7 days of DSS induction of acute colitis, concomitant with PIN or 5-ASA) and rats were divided into five groups (*n* = 6 each), (1) control group, (2) DSS colitis group, (3) PIN low dosage group (5 mg/kg b.w., *p.o.*), (4) PIN high dosage group (10 mg/kg b.w., *p.o.*), and (5) 5-ASA group (reference drug, 50 mg/kg b.w., *p.o.*). PIN and 5-ASA group rats received drugs for 7 days, prior to the DSS treatment and treated until the last day of the experiment. DSS was added in the drinking water for 7 days at 3.5% (w/v) for acute colitis induction. The colitis severity was evaluated from the DSS induction, based on the disease activity index (DAI), with the loss of body weight, stool consistency, rectal bleeding, and the overall health condition (Wang et al., 2017).

### Histological Evaluation on the Distal Colon

All animals were anesthetized and sacrificed at the final day of the experiment. Distal colon samples were collected and fixed distal colon samples with neutral buffered formalin embedded in paraffin and stained with hematoxylin-eosin (HE) or periodic acid–Schiff (PAS) for histopathology examination. After deparaffinization and hydration, colonic sections were immersed in 1% periodic acid for 8 min, then placed in Schiff’s reagent for 15 min, followed by dehydration, clearance and mounting in Canada balsam (Balaha et al., 2016). Goblet cell distribution was observed from 5 randomly selected crypts per colon using PAS-stained cross sections. Colonic tight junction proteins (ZO-1 and Occludin) were examined by immunohistochemical staining method. Paraffin sections were heated at 60° for 1 h in an oven. The de-paraffinization, endogenous peroxidase was blocked by 3% H_2_O_2_ for 15 min, and the sections were incubated in primary antibody of ZO-1 and Occludin (Proteintech Group, Chicago, IL, United States) overnight at 4° in the refrigerator. After washed, the sections were incubated in secondary antibody for 15 min. Next, freshly prepared diaminobenzidine (DAB) solution was applied to visualize antibody, followed by hematoxylin staining, dehydration and mounting. The light microscope attached imaging system (Nikon Eclipse Ci, Japan) was used to visualize the stained slides and acquire images. Colonic sections were coded for blind microscopic assessment of histological changes by two pathologists. Colonic histological damage was scored from H&E-stained slides based on two subscores (cell infiltration and tissue damage), ranging from 0 to 6 (no changes to extensive cell infiltration and tissue damage). IHC score were calculated previously by using the formula: IHC score = Σ(I × Pi), where I, intensity of staining and Pi, percentage of ZO-1 stained colonic cells, ranging from 0 to 300 (Yeo et al., 2015).

### Transmission Electron Microscopy (TEM)

Distal colon samples were fixed in glutaraldehyde (2.5%) and 1% osmium tetroxide (1%) in 0.01 M phosphate buffer (pH 7.0). Samples were then embedded in Epon 812 resin overnight and dried with gradient acetone, following the manufacturer’s instructions (SPI-EM, Division of Structure Probe, Westchester, NY, United States). After obtaining ultrathin sections (70 nm), uranium acid and lead citrate were used for drying, and samples were observed under a transmission electron microscopy (TEM, Hitachi, Tokyo, Japan).

### RNA Extraction and Quantitative Real-Time PCR (qPCR)

RNA was extracted from distal colon using a commercial kit (Carry Helix Biotechnologies Co., Ltd., Beijing, China) and synthesized to cDNA by reverse transcription (TaKaRa, Dalian, China). Quantitative real-time PCR was performed using a two-step amplification method and all information was collected by under the 7500c Real-time PCR Detection System (Applied Biosystems, Carlsbad, CA, United States) and calculated the transcriptional levels of target genes using the 2^–△△*Ct*^ method based on cycle threshold (Ct) values and *GAPDH* was served as the housekeeping gene. Specific primers sequences can be found in our previous published literature (Wang et al., 2016a, 2018).

### Short Chain Fatty Acids (SCFA) Analysis

Cecal digesta (∼150 mg) collected from rats were weighed and diluted at 1:3 (w/w) with deionized water containing 1.68 mM heptanoic acid/L as an internal standard (Sigma Chemical Co., St. Louis, MO, United States). Acetate, butyrate, propionate, and the total SCFA (including minor SCFA) were measured using a gas chromatography (GC) system based on our previous published methods (Wang et al., 2017).

### Gut Microbial Community Analysis

The V3-V4 region of the 16S rRNA gene of the caecal microorganism was amplified after extracting DNA for pyrosequencing (Qiagen, Hilden, Germany). Universal primers: 319F (5′-ACTCCTACGGGAGGCAGCAG-3′) and 806R (5′-GGACTACHVGGGTWTCTAAT-3′) were applied for bacterial gene amplification using Premix Ex TaqTM Hot Start (Takara, Dalian, China). Then PCR products were sequenced using MiSeq Illumina sequencing (Illumina, United States) and the software package QIIME (version 1.17). Then UPARSE (version 7.1) software was applied to cluster the sequences and obtained operational taxonomic units (OTUs) with 97% similarity. Bacterial taxonomic profiles at different levels were generated within QIIME using the RDP classifier against the SILVA (SSU115) 16S rRNA database with a confidence threshold of 70% using the default settings. A Venn diagram was generated based on the relative abundances of OTUs in each sample from three groups (Normal, DSS and PVH treatment). Beta-diversity was measured by principal coordinate analysis (PCoA) at the OTU level and hierarchical clustering tree on Genus level was calculated based on UniFrac metrics dissimilarity matrix using Vegan 2.0 packages in the R (version 3.1.2) statistical environment.

### Statistical Analysis

Data are presented as the arithmetic mean ± SD for indicated replicates. The effect of treatments was determined by one-way ANOVA and differences between treatments were analyzed *post hoc* by Tukey’s honest significant difference test (*p*-values ≤0.05 were considered statistically significant) using SPSS version 17.0.

## Results

### PIN Alleviated DSS-Induced Acute Colitis in Rats

We observed the potential protective effects of PIN against colonic inflammation, DSS was used to induce the acute colitis to the rats for 7 days, followed by 1 week of oral pre-administration with different doses of PIN (5 and 10 mg/kg) and 5-ASA (50 mg/kg, reference drug). As shown in Figure 1B, DSS-treated rats exhibited severe colitis symptoms, marked by shortened, thickened and erythematous colons, which were attenuated in PIN or 5-ASA treated rats. Figure 1C shows significant decrease of body weight gains in the DSS-induced colitis rats (61.8 ± 7.5 g in control group vs. 27.3 ± 3.3 in DSS group). Body weight data also suggested that rats in high dosage of PIN and 5-ASA administrations have less body weight loss compared with DSS control group. We also noticed that DSS control groups had severe colonic damages and disease activity index (DAI) among the groups (Figure 1D), compared with DSS control, PIN treatment regulated the DAI index (Figure 1D), and also prevented reduction in colon length/body weight ratio (Figure 1E). The protective effects in high dosage PIN group are even comparable to that of the 5-ASA group.

### Effect of PIN Treatment on the Colonic Pathological Changes and Goblet Cells in DSS-Induced Colitis Rats

**FIGURE 2:**
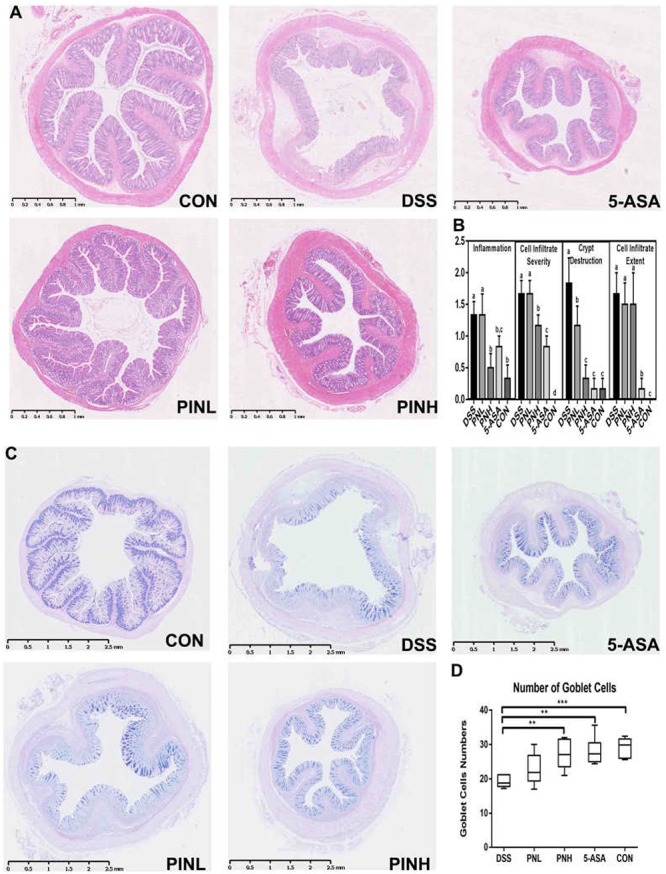
Pinocembrin pretreatment relieved the colonic pathological changes and loss of goblet cells in DSS-induced colitis rats. **(A)** The distal colon sections were stained with hematoxylin-eosin (HE). **(B)** Histological scores of colon sections from each group and scores are expressed as means ± SD (*n* = 8 for each group). Groups with different letters differ by a statistically significant margin (*p* < 0.05). **(C)** The distal colon sections were subjected to Periodic Acid-Schiff (PAS) staining of goblet cells (blue) in the colon. **(D)** Averages of goblet cells/crypt in the colon are shown. The data represent the mean ± SD of six rats in each group. The stars indicate that value was significantly different (^∗∗^*p* < 0.01, ^∗∗∗^*p* < 0.001) compared with DSS control.

Severe histological colonic damages were observed in the DSS-induced colitis rats group due to infiltration of immune cells and muscle layer thickness (Figure 2A). Histopathological analysis suggested that pre-treated rats with PIN or 5-ASA showed near normal control histology of the colonic epithelium, compared with DSS induced rats (Figure 2B). We also observed PAS staining of the goblet cells (Figure 2C), found decreased goblets cells in DSS-induced colitis colon rats (19.2 ± 1.6 per crypts), and whereas PIN treated rats showed regulated goblet cells (22.8 ± 4.6, low dosage, and 27.2 ± 4.5 per crypts, high dosage) near to normal control rats (29.2 ± 2.9 per crypts Figure 2D).

### Effect of PIN Treatment on Colonic mRNA Expressions in DSS-Induced Colitis Rats

**FIGURE 3:**
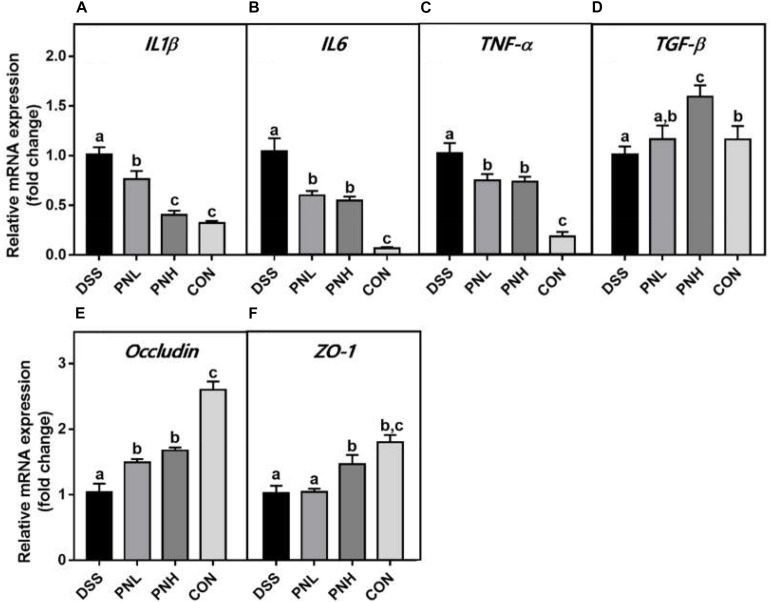
Effect of pinocembrin on colonic mRNA expressions in DSS-induced colitis rats. Distal colons of the rats were collected and mRNA expression of IL-1β **(A)** IL-6 **(B)** TNF-α **(C)** TGF-β **(D**) Occludin **(E)** and ZO-1 **(F)** was quantified using real-time PCR. Fold changes are expressed as means ± SD (*n* = 8 for each group in a single experiment). Groups with different letters differ by a statistically significant margin (*p* < 0.05).

As shown in Figure 3, pretreatment with PIN regulated the anti-inflammatory responses mediated by colonic *TGF-β* expression and mRNA expression of the pro-inflammatory mediators such as *IL-1β*, *IL-6*, and *TNF-α* in DSS-induced rats. Moreover, increased gene expressions of tight junction proteins, ZO-1 and Occludin were observed in PIN pre-administrated groups, which benefits the mucosal barrier function and epithelial restitution.

### PIN Improved the Tight Junction Functions in DSS-Induced Colitis Rats

**FIGURE 4:**
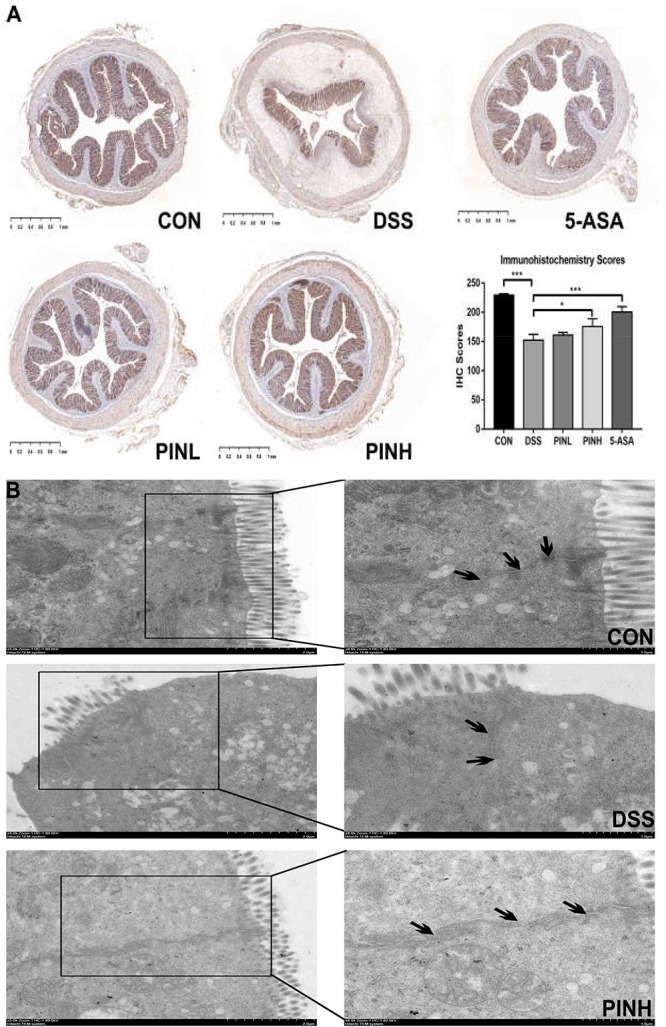
Effects of pinocembrin on colon tight junction. Distal colon was collected and embedded in paraffin for ZO-1 immunohistochemistry **(A)** or fixed in glutaraldehyde for transmission electron micrographs (TEM, **B**). Black arrows indicated tight junctions between the colonic epithelial cells. The immunohistochemistry scores of ZO-1 are expressed as the mean ± SD of four rats in each group. The stars indicate that values were significantly different (^*^p < 0.05, ^∗∗∗^*p* < 0.001) compared with DSS control.

As shown in Figure 4A, we observed the DSS group showed decreased ZO-1 expressions in their distal colon mucosa. PIN pre-treatment (7 days) reverted the loss of TJ compared with DSS induced rats using IHC method. TEM analysis suggested that PIN pretreatment restored the enlarged TJ and expansion of the endoplasmic reticulum compared with DSS-induced rats (Figure 4B).

### Effect of PIN on Cecal SCFAs and Microbiota Composition in DSS-Induced Colitis Rats

**FIGURE 5:**
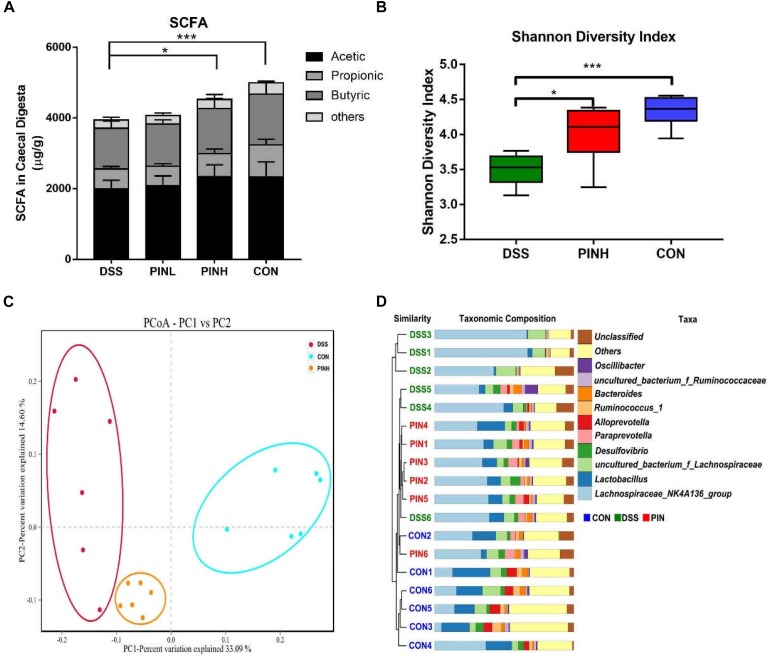
Effect of pinocembrin on intestinal short chain fatty acid (SCFA) and gut microbiota composition in DSS-induced colitis rats. Rats were treated as described same above and cecal digesta were collected. **(A)** SCFA was measured using a gas chromatography (GC). Compound information represented by different colors is marked in the upper right. **(B)** Cecal microbial communities were measured using 16S rRNA sequencing. Alpha diversity of rats cecal microbiota in each group was determined by Shannon index (^*^p < 0.05, ^∗∗∗^*p* < 0.001). **(C)** Beta diversity of rats cecal microbiota in each group was determined by Principal coordinate analysis (PCoA) of weighted UniFrac distance. **(D)** Unweighted Pair Group Method with Arithmetic Mean (UPGMA) clustering of unweighed UniFrac β-diversity distances showing microbiota taxa composition at the genus level. Bacteria represented by different colors are marked in the upper right.

We next measured the cecal SCFA concentrations, including acetate, propionate, butyrate, and total SCFA by GC, which indicated as hallmarks for intestinal microbial metabolic products. Total SCFA concentrations as well as acetate, propionate and butyrate were similarly decreased in DSS colitis rats (3943.8 ± 105.6 μg/g), compared with normal control rats (4666.9 ± 213.8 μg/g). High dosage of PIN pre-administration resulted in significant increase of SCFA (4521.1 ± 174.2 μg/g), indicating its beneficial modifications on the colonic microbial populations during colitis (Figure 5A).

As high dosage of PIN showed best gastrointestinal protective effects, we next chose this group together with control and DSS groups, to analysis cecal microbial community structure using 16S ribosomal RNA Illumina next-generation sequencing. We observed a significant decrease of the Shannon α-diversity index between DSS-induced and control rats.PIN pretreatment showed an overall impact on increasing the Shannon index (Figure 5B). Furthermore, we noticed a significant difference in microbial β-diversity, based on Principal coordinate analysis (PCoA) of weighed UniFrac distance (Figure 5C). Unweighted Pair Group Method with Arithmetic Mean (UPGMA) was further used to show Unifrac alpha and beta diversity at the genus level. We noticed that DSS has less diversity of bacteria genera, compared with the PIN and control groups. Some specific bacteria genera, including *Lactobacillus* spp., *Alloprevotella* spp., and *Desulfovibrio* spp. were rescued by PIN (Figure 5D).

## Discussion

In the previous studies, we found that Chinese propolis administrations showed protective effects against acute colitis symptoms using rodent models and the intestinal mucosal barrier functions were improved in human intestinal cells (Wang et al., 2016a, 2018). As Chinese propolis contains abundant polyphenolic constitutions, like (CAPE, chrysin, galanin, and PIN)(Huang et al., 2014), we further showed that PIN might be a key contributor for the anti-colitis and modulating the gut microbiota. We used DSS-induced colitis in rats, which is a well-known experimental model and mimics clinical symptoms similar to UC (Wang et al., 2018). In agreement with previous reports, significant reduced body weight gains, a dramatic increase of DAI accompany with shortening the length of colon was observed in DSS-induced rats and suggesting the successful repentance of the model we used (Bramhall et al., 2015; Munyaka et al., 2016). We chose the dose of PIN at 5 and 10 mg/kg (∼5 and 10-fold of the human dose), *p.o.*, based on several previous publications which support the safe usage of PIN in rodents (Gao et al., 2010; Liu et al., 2014). We showed PIN at the dose of 10 mg/kg had comparable protective effects to 5-ASA against colitis symptoms, a reference drug which was most widely used in clinical practice (Wang et al., 2016b). We also showed PIN pretreated rats histopathological damages were near normal control rats and the DSS induced colitis rats showed thick colonic mucus layer, which indicates that a loss of function in the physical barrier between the epithelium and lumen (Sperandio et al., 2015). Since colonic mucus is mainly secreted from the goblet cells, an increase of goblet cell numbers by PIN indicated that a restored mucous barrier in the gastrointestinal tract (Johansson and Hansson, 2016).

It has been recognized that the integrate mucosal barrier is important for maintaining the hemostasis of the gut, since the leaky gut barrier facilitates the invading of pathological bacteria into the colonic mucosa, lead to system innate and inflammatory immune responses in the host (Yang et al., 2017). Recent studies suggested that the increase of pro-inflammatory cytokine, TNF-α, lead to intestinal dysfunction along with the other pro-inflammatory cytokines such as IL-1β and IL-6 (Zhang et al., 2018a). Consistent with previous findings in macrophages and microglial cells (Soromou et al., 2012; Zhou et al., 2015), PIN pre-administration showed strong anti-inflammatory effects against the inflammation-related gene expressions. The TGF-β is one of an anti-inflammatory cytokine which showed alleviating the colitis and protecting the intestinal epithelial cells. It is required for intestinal mucosal healing, and lack of TGF-β strongly increases the epithelial susceptibility to injury (Beck et al., 2003; Bermudez-Humaran et al., 2015). In the present study, the PIN increased the TGF-β expression in colitis rats, which was consistent with a previous study (Zhou et al., 2018). The gut physiological barrier between the intestinal epithelial cells is formed by the TJ complex, which has key roles in restricting and modulating the intestinal permeability. Several transmembrane proteins and cytosolic adaptor proteins composed the TJ. Occludin is an integral plasma membrane protein and zona occludens (ZO) proteins-1 a peripheral membrane protein, which are indicators for tight junction assembly, stability, and barrier function (Zihni et al., 2016). Previous studies suggested that several intestinal pathologies are associated with TJ barrier disruptions, including IBD (Suzuki and Hara, 2011). A number of dietary nutrients, including flavonoids, have been demonstrated to increase TJ function as well as TJ proteins (Gil-Cardoso et al., 2016; Yang et al., 2017). Such natural flavonoids including quercetin (Suzuki and Hara, 2009), galangin (Zhang et al., 2019), naringenin (Dou et al., 2013). Inconsistent with these studies, pre-administration of PIN regulated the gene and protein expressions of ZO-1 and Occludin in the colon. Electron microscopy images clearly showed that PIN dampened wide intercellular spaces and the damaged tight TJ structures in colitis rats (Zhang et al., 2018b). Nevertheless, the molecular mechanisms of regulating effects of TJs by PIN still need to be explored clearly. The gastrointestinal microenvironment is a complex orchestration between the host and the gut microbial populations. We showed that cecal SCFA levels were decreased significantly by DSS, which were consistent with previous findings (Wu et al., 2018). We demonstrated that PIN increased SCFA concentrations (acetate and butyrate) in colitis rats, indicative of increased microbial activity of the gut (Monk et al., 2016). Clinical evidence showed that IBD patients lead to alterations in the diversity and composition of their gut microbiota. Dietary intervention recently has been attracted much attention for the potential applications into complementary/alternative approaches of IBD prevention or treatment (Yin et al., 2018a, b).

Our studies showed that dietary intake of PIN rich propolis has great protective effects against DSS-induced rats colitis, and modulates gut microbiota composition as well as increases the intestinal microflora diversity. Although there is scant of studies investigated the modulating effects on the gut microbiota of PIN, we showed that PIN reversing the decrease on the gut microflora community induced by DSS, based on the alpha diversity of the Shannon index (Cui et al., 2018). The decrease in the probiotic bacteria, like *Lactobacillus* spp. was noticed from DSS-induced rats, while PIN reversed the alteration. The modulations of the gut microflora could also potentially explain the protective effects by PIN against DSS-induced rats colitis.

## Conclusion

Overall, we observed that the oral pretreatment of PIN, a natural flavonoid, showed significant protective effects against acute colitis induced by DSS. These beneficial effects might be attributed to the anti-inflammatory effects, restoring on the intestinal barrier function and modulating on the gut microbiota. Our study provides important pre-clinical evidence for the potential application of PIN to the prevention or treatment of gastrointestinal disorders. However, further studies are needed to identify the exact underlying mechanisms of PIN consumption against IBDs.

## Data Availability

The raw data supporting the conclusions of this manuscript will be made available by the authors, without undue reservation, to any qualified researcher.

## Ethics Statement

This study was carried out in accordance with the recommendations of the ARRIVE (Animal Research: Reporting of *In vivo* Experiments) guidelines, Animal Ethics Committee of Institute of Apicultural Research, and the Chinese Academy of Agricultural Sciences. The protocol was approved by the Animal Ethics Committee of Institute of Apicultural Research and the Chinese Academy of Agricultural Sciences.

## Author Contributions

LH and KW carried out the study and performed the statistical analysis. CW, ML, and ZZ provided help in performing the experiments. KW and YC designed the research. LH, KW, and YC prepared the draft of the manuscript. EM read and revised the final version of the manuscript.

## Conflict of Interest Statement

The authors declare that the research was conducted in the absence of any commercial or financial relationships that could be construed as a potential conflict of interest.

## References

[B1] Ananthakrishnan A. N. (2015). Epidemiology and risk factors for IBD. *Nat. Rev. Gastroenterol. Hepatol.* 12 205–217. 10.1038/nrgastro.2015.34 25732745

[B2] Azad M. A. K. Sarker M. Li T. Yin J. (2018). Probiotic species in the modulation of gut microbiota: an overview. *Biomed Res. Int.* 2018:9478630. 10.1155/2018/9478630 29854813 PMC5964481

[B3] Balaha M. Kandeel S. Elwan W. (2016). Garlic oil inhibits dextran sodium sulfate-induced ulcerative colitis in rats. *Life Sci.* 146 40–51. 10.1016/j.lfs.2016.01.012 26780265

[B4] Beck P. L. Rosenberg I. M. Xavier R. J. Koh T. Wong J. F. Podolsky D. K. (2003). Transforming growth factor-beta mediates intestinal healing and susceptibility to injury in vitro and in vivo through epithelial cells. *Am. J. Pathol.* 162 597–608. 10.1016/s0002-9440(10)63853-9 12547717 PMC1851153

[B5] Bermudez-Humaran L. G. Motta J. P. Aubry C. Kharrat P. Rous-Martin L. Sallenave J. M. (2015). Serine protease inhibitors protect better than IL-10 and TGF-beta anti-inflammatory cytokines against mouse colitis when delivered by recombinant lactococci. *Microb. Cell Fact.* 14:26. 10.1186/s12934-015-0198-4 25889561 PMC4371826

[B6] Bramhall M. Florez-Vargas O. Stevens R. Brass A. Cruickshank S. (2015). Quality of methods reporting in animal models of colitis. *Inflamm. Bowel Dis.* 21 1248–1259. 10.1097/MIB.0000000000000369 25989337 PMC4450905

[B7] Cao H. Liu J. Shen P. Cai J. Han Y. Zhu K. (2018). Protective effect of naringin on DSS-induced ulcerative colitis in mice. *J. Agric. Food Chem.* 66 13133–13140. 10.1021/acs.jafc.8b03942 30472831

[B8] Cui H. Cai Y. Wang L. Jia B. Li J. Zhao S. (2018). Berberine regulates Treg/Th17 balance to treat ulcerative colitis through modulating the gut microbiota in the colon. *Front. Pharmacol.* 9:571. 10.3389/fphar.2018.00571 29904348 PMC5991375

[B9] Dou W. Zhang J. Sun A. Zhang E. Ding L. Mukherjee S. (2013). Protective effect of naringenin against experimental colitis via suppression of Toll-like receptor 4/NF-kappaB signalling. *Br. J. Nutr.* 110 599–608. 10.1017/S0007114512005594 23506745 PMC3726555

[B10] Gao M. Zhu S. Y. Tan C. B. Xu B. Zhang W. C. Du G. H. (2010). Pinocembrin protects the neurovascular unit by reducing inflammation and extracellular proteolysis in MCAO rats. *J. Asian Nat. Prod. Res.* 12 407–418. 10.1080/10286020.2010.485129 20496198

[B11] Gil-Cardoso K. Gines I. Pinent M. Ardevol A. Blay M. Terra X. (2016). Effects of flavonoids on intestinal inflammation, barrier integrity and changes in gut microbiota during diet-induced obesity. *Nutr. Res. Rev.* 29 234–248. 10.1017/S0954422416000159 27841104

[B12] Huang S. Zhang C. P. Wang K. Li G. Q. Hu F. L. (2014). Recent advances in the chemical composition of propolis. *Molecules* 19 19610–19632. 10.3390/molecules191219610 25432012 PMC6271758

[B13] Johansson M. E. Hansson G. C. (2016). Immunological aspects of intestinal mucus and mucins. *Nat. Rev. Immunol.* 16 639–649. 10.1038/nri.2016.88 27498766 PMC6435297

[B14] Lan X. Wang W. Li Q. Wang J. (2016). The natural flavonoid pinocembrin: molecular targets and potential therapeutic applications. *Mol. Neurobiol.* 53 1794–1801. 10.1007/s12035-015-9125-2 25744566 PMC4561606

[B15] Lane E. R. Zisman T. L. Suskind D. L. (2017). The microbiota in inflammatory bowel disease: current and therapeutic insights. *J. Inflamm. Res.* 10 63–73. 10.2147/JIR.S116088 28652796 PMC5473501

[B16] Liu R. Li J. Z. Song J. K. Zhou D. Huang C. Bai X. Y. (2014). Pinocembrin improves cognition and protects the neurovascular unit in Alzheimer related deficits. *Neurobiol. Aging* 35 1275–1285. 10.1016/j.neurobiolaging.2013.12.031 24468471

[B17] Martin D. A. Bolling B. W. (2015). A review of the efficacy of dietary polyphenols in experimental models of inflammatory bowel diseases. *Food Funct.* 6 1773–1786. 10.1039/c5fo00202h 25986932

[B18] Monk J. M. Lepp D. Zhang C. P. Wu W. Zarepoor L. Lu J. T. (2016). Diets enriched with cranberry beans alter the microbiota and mitigate colitis severity and associated inflammation. *J. Nutr. Biochem.* 28 129–139. 10.1016/j.jnutbio.2015.10.014 26878790

[B19] Moura F. A. de Andrade K. Q. Dos Santos J. C. F. Araujo O. R. P. Goulart M. O. F. (2015). Antioxidant therapy for treatment of inflammatory bowel disease: does it work? *Redox Biol.* 6 617–639. 10.1016/j.redox.2015.10.006 26520808 PMC4637335

[B20] Munyaka P. M. Rabbi M. F. Khafipour E. Ghia J. E. (2016). Acute dextran sulfate sodium (DSS)-induced colitis promotes gut microbial dysbiosis in mice. *J. Basic Microbiol.* 56 986–998. 10.1002/jobm.201500726 27112251

[B21] Neurath M. (2017). Current and emerging therapeutic targets for IBD. *Nat. Rev. Gastroenterol. Hepatol.* 14 269–278. 10.1038/nrgastro.2017.138 28144028

[B22] Peng L. Yang S. Cheng Y. J. Chen F. Pan S. Fan G. (2012). Antifungal activity and action mode of pinocembrin from propolis against Penicillium italicum. *Food Sci. Biotechnol.* 21 1533–1539. 10.1007/s10068-012-0204-0

[B23] Rasul A. Millimouno F. M. Ali Eltayb W. Ali M. Li J. Li X. (2013). Pinocembrin: a novel natural compound with versatile pharmacological and biological activities. *Biomed Res. Int.* 2013:379850. 10.1155/2013/379850 23984355 PMC3747598

[B24] Soromou L. W. Chu X. Jiang L. Wei M. Huo M. Chen N. (2012). In vitro and in vivo protection provided by pinocembrin against lipopolysaccharide-induced inflammatory responses. *Int. Immunopharmacol.* 14 66–74. 10.1016/j.intimp.2012.06.009 22713932

[B25] Sperandio B. Fischer N. Sansonetti P. J. (2015). Mucosal physical and chemical innate barriers: lessons from microbial evasion strategies. *Semin. Immunol.* 27 111–118. 10.1016/j.smim.2015.03.011 25936225

[B26] Suzuki T. Hara H. (2009). Quercetin enhances intestinal barrier function through the assembly of zonula [corrected] occludens-2, occludin, and claudin-1 and the expression of claudin-4 in Caco-2 cells. *J. Nutr.* 139 965–974. 10.3945/jn.108.100867 19297429

[B27] Suzuki T. Hara H. (2011). Role of flavonoids in intestinal tight junction regulation. *J. Nutr. Biochem.* 22 401–408. 10.1016/j.jnutbio.2010.08.001 21167699

[B28] Wang K. Jin X. Chen Y. Song Z. Jiang X. Hu F. (2016a). polyphenol-rich propolis extracts strengthen intestinal barrier function by activating AMPK and ERK Signaling. *Nutrients* 8:E272. 10.3390/nu8050272 27164138 PMC4882685

[B29] Wang Y. Parker C. E. Feagan B. G. MacDonald J. K. (2016b). Oral 5-aminosalicylic acid for maintenance of remission in ulcerative colitis. *Cochrane Database Syst. Rev.* 9:CD000544. 10.1002/14651858.CD000544.pub4 27158764 PMC7045447

[B30] Wang K. Jin X. Li Q. Sawaya A. Le Leu R. K. Conlon M. A. (2018). Propolis from different geographic origins decreases intestinal inflammation and *Bacteroides* spp. populations in a model of DSS-induced colitis. *Mol. Nutr. Food Res.* 62:e1800080. 10.1002/mnfr.201800080 29889351

[B31] Wang K. Jin X. You M. Tian W. Le Leu R. K. Topping D. L. (2017). Dietary propolis ameliorates dextran sulfate sodium-induced colitis and modulates the gut microbiota in rats fed a western diet. *Nutrients* 9:875. 10.3390/nu9080875 28805735 PMC5579668

[B32] Wu X. Wang L. Tang L. Wang L. Cao S. Wu Q. (2018). Salvianolic acid B alters the gut microbiota and mitigates colitis severity and associated inflammation. *J. Funct. Foods* 46 312–319. 10.1016/j.jff.2018.04.068

[B33] Yang G. Bibi S. Du M. Suzuki T. Zhu M. J. (2017). Regulation of the intestinal tight junction by natural polyphenols: a mechanistic perspective. *Crit. Rev. Food Sci. Nutr.* 57 3830–3839. 10.1080/10408398.2016.1152230 27008212

[B34] Yeo W. Chan S. L. Mo F. K. Chu C. M. Hui J. W. Tong J. H. (2015). Phase I/II study of temsirolimus for patients with unresectable Hepatocellular Carcinoma (HCC)- a correlative study to explore potential biomarkers for response. *BMC Cancer* 15:395. 10.1186/s12885-015-1334-6 25962426 PMC4434865

[B35] Yin J. Li Y. Han H. Chen S. Gao J. Liu G. (2018a). Melatonin reprogramming of gut microbiota improves lipid dysmetabolism in high-fat diet-fed mice. *J. Pineal Res.* 65:e12524. 10.1111/jpi.12524 30230594

[B36] Yin J. Li Y. Han H. Liu Z. Zeng X. Li T. (2018b). Long-term effects of lysine concentration on growth performance, intestinal microbiome, and metabolic profiles in a pig model. *Food Funct.* 9 4153–4163. 10.1039/c8fo00973b 30058657

[B37] Zhang H. Hua R. Zhang B. Zhang X. Yang H. Zhou X. (2018a). Serine alleviates dextran sulfate sodium-induced colitis and regulates the gut microbiota in mice. *Front. Microbiol.* 9:3062. 10.3389/fmicb.2018.03062 30619148 PMC6295577

[B38] Zhang Y. Zhao X. Zhu Y. Ma J. Ma H. Zhang H. (2018b). Probiotic Mixture protects dextran sulfate sodium-induced colitis by altering tight junction protein expressions and increasing tregs. *Mediators Inflamm* 2018:9416391. 10.1155/2018/9416391 29849501 PMC5925202

[B39] Zhang T. Mei X. Ouyang H. Lu B. Yu Z. Wang Z. (2019). Natural flavonoid galangin alleviates microglia-trigged blood-retinal barrier dysfunction during the development of diabetic retinopathy. *J. Nutr. Biochem.* 65 1–14. 10.1016/j.jnutbio.2018.11.006 30597356

[B40] Zhou F. Wang A. Li D. Wang Y. Lin L. (2018). Pinocembrin from penthorum chinense pursh suppresses hepatic stellate cells activation through a unified SIRT3-TGF-beta-Smad signaling pathway. *Toxicol. Appl. Pharmacol.* 341 38–50. 10.1016/j.taap.2018.01.009 29352975

[B41] Zhou L. T. Wang K. J. Li L. Li H. Geng M. (2015). Pinocembrin inhibits lipopolysaccharide-induced inflammatory mediators production in BV2 microglial cells through suppression of PI3K/Akt/NF-kappaB pathway. *Eur. J. Pharmacol.* 761 211–216. 10.1016/j.ejphar.2015.06.003 26049009

[B42] Zihni C. Mills C. Matter K. Balda M. S. (2016). Tight junctions: from simple barriers to multifunctional molecular gates. *Nat. Rev. Mol. Cell Biol.* 17 564–580. 10.1038/nrm.2016.80 27353478

